# Design and Characterization of Paclitaxel-Loaded Polymeric Nanoparticles Decorated With Trastuzumab for the Effective Treatment of Breast Cancer

**DOI:** 10.3389/fphar.2022.855294

**Published:** 2022-03-14

**Authors:** Mirina Sakhi, Abad Khan, Zafar Iqbal, Ismail Khan, Abida Raza, Asmat Ullah, Fazli Nasir, Saeed Ahmad Khan

**Affiliations:** ^1^ Department of Pharmacy, University of Swabi, Swabi, Pakistan; ^2^ Department of Pharmacy, University of Peshawar, Peshawar, Pakistan; ^3^ National Institute of LASER and Optronics, Nilore, Pakistan; ^4^ Department of Pharmacy, Kohat University of Science and Technology, Kohat, Pakistan

**Keywords:** biodegradable, polymeric, drug-delivery, breast cancer, paclitaxel, cytotoxicity, trastuzumab, PLGA

## Abstract

The aim of the study was to design and formulate an antibody-mediated targeted, biodegradable polymeric drug delivery system releasing drug in a controlled manner to achieve a therapeutic goal for the effective treatment of breast cancer. Antibody-mediated paclitaxel-loaded PLGA polymeric nanoformulations were prepared by the solvent evaporation method using different experimental parameters and compatibility studies. The optimized formulations were selected for *in vitro* and *in vivo* evaluation and cytotoxicity studies. The *in vitro* drug release studies show a biphasic release pattern for the paclitaxel-loaded PLGA nanoparticles showing a burst release for 24 h followed by an extended release for 14 days; however, a more controlled and sustained release was observed for antibody-conjugated polymeric nanoparticles. The cytotoxicity of reference drug and paclitaxel-loaded PLGA nanoparticles with and without antibody was determined by performing MTT assay against MCF-7 cells. Rabbits were used as experimental animals for the assessment of various *in vivo* pharmacokinetic parameters of selected formulations. The pharmacokinetic parameters such as C_max_ (1.18–1.33 folds), AUC_0-t_ (39.38–46.55 folds), MRT (10.04–12.79 folds), t_1/2_ (3.06–4.6 folds), and V_d_ (6.96–8.38 folds) have been increased significantly while clearance (4.34–4.61 folds) has been decreased significantly for the selected nanoformulations as compared to commercially available paclitaxel formulation (Paclixil^®^). The surface conjugation of nanoparticles with trastuzumab resulted in an increase in *in vitro* cytotoxicity as compared to plain nanoformulations and commercially available conventional brand (Paclixil^®^). The developed PLGA-paclitaxel nanoformulations conjugated with trastuzumab have the desired physiochemical characteristics, surface morphology, sustained release kinetics, and enhanced targeting.

## 1 Introduction

Cancer is a disease in which genes regulating the functions of cells, i.e., cell growth, division, differentiation, and cell death losses are without any control (Liotta et al., 1991). Cancer is developing very rapidly in the whole world, especially in the developing countries. In women, among all the cancers diagnosed, breast cancer accounts for one-third (Miele et al., 2009), and 18.2% of deaths are caused by breast cancer worldwide. Breast cancer receptors are divided into two main types, i.e., estrogen receptor (ER) negative and human epidermal growth factor receptors (HER2) positive (Carey et al., 2006). Breast cancer is treated nowadays by different ways, i.e., hormone-blocking agents, chemotherapy, radiotherapy, monoclonal antibodies, and surgery (Waks & Winer, 2019).

Main problems with concventional drug delivery systems are fluctuations of drug concentrations in blood which in turn causes subtherapeutic concentration or toxic effects. Lack of specificity, multidrug resistance, toxicity of chemotherapeutic agents, side effects, limited aqueous solubility, and poor bioavailability are some of the limitations with available cancer therapy (Chidambaram et al., 2011). The major target of any drug delivery system and particularly controlled drug delivery system is to make the therapeutically effective amount of drug available at a desired site, at an optimum concentration, and for a desired period of time (Win, 2006).

Nanotechnology is gaining much popularity as mortality due to cancer continues to rise, and the advanced nanotechnology has provided an effective approach for targeting the drug to tumor tissues by overcoming the limitations that are associated with conventional chemotherapeutic agents (Ferlay et al., 2015). Nanotechnology has shown a new path for the development of various organic and inorganic drug carriers called as nanoparticles.

Biodegradable polymers are the first choice in nanoparticulate drug delivery because they not only release drugs in a controlled manner but are also compatible with tissues and cells (Fonseca et al., 2002). In the last 10–20 years, the polymeric biodegradable nanoparticle drug delivery has got a lot of importance in cancer treatments. Among these polymers, one of the biodegradable polymers used most successfully is poly lactic co glycolic acid (PLGA) which upon hydrolysis is metabolized to lactic and glycolic acid and excreted quickly (Kumari et al., 2010). PLGA has been approved by the Food and Drug Administration (FDA) for parenteral administrations due to its biodegradability and biocompatibility. It can be easily formulated with a variety of hydrophilic or hydrophobic molecules, and it imparts some extra properties to the drug molecules, i.e., protect drug from degradation effects, control the release, and can also modify the surface in order to interact with other biological materials and to achieve stealth or targeted delivery of nanoparticles (Danhier et al., 2012).

Poloxamer 407 is a cationic, tri-block copolymer containing polyethylene oxide (hydrophilic portion) and poly propylene oxide (hydrophobic portion). The hydrophobic end is anchored with the nanoparticle surface, while the water loving portion is toward the aqueous medium forming a hydrophilic layer (Redhead et al., 2001; Stolnik et al., 2001). It is amphiphilic in nature with bioadhesive properties and increases solubilization of hydrophobic drugs. Poloxamer 407 has been approved by the FDA as a bioactive ingredient for topical, ophthalmic, suspension, injectable, and other pharmaceutical preparations (Dumortier et al., 2006). Nanoparticles, whose surfaces have been modified with poloxamer 407 remain in blood circulation for a prolonged period of time, escapes the reticuloendothelial system (Stolnik et al., 2001). Poloxamer 407 increases drug accumulation inside tumor tissue by inhibiting the efflux transport protein system. This provides steric stabilization by inhibiting phagocytosis and prevention of protein adsorption (Moura et al., 2020). Poloxamer 407 enhances bioavailability by increasing drug residence time (Moghimi & Hunter, 2000). New therapeutic strategies can be developed using poloxamer because of its temperature-dependent self-assembly characteristic. It can be used for increasing the stability and solubility of drugs (Carvalho et al., 2021).

The HER family of receptors are of prime importance in the pathogenesis of several cancers by regulating the cell differentiation, growth, and survival through multiple pathways (Romond et al., 2005). This family of receptors is made up of four main members: HER (1, 2, 3, and 4) or Erb (B1, B2, B3, and B4). All four HERs consist of an intracellular and extracellular binding site (Sun et al., 2011b; Iqbal & Iqbal, 2014). Monoclonal antibodies are clones of a unique parent cell and recognize specific antigens that are located on the cancer cell surface, thereby causing an antigen–antibody-like effect through multiple mechanisms which include ligand–receptor binding interference or protein expression suppression (Steichen et al., 2013). Improved clinical efficacy and decreased toxicity associated with conventional anticancer drugs attributed to the significant use of monoclonal antibodies (Colzani et al., 2018). Trastuzumab, a humanized monoclonal antibody approved by the US-FDA for breast cancer, targets overexpressed HER2 receptors in breast cancer cells. Combination therapy of trastuzumab with conventional chemotherapeutics leads to increased response rates in comparison to trastuzumab alone (Piccart-Gebhart et al., 2005; P.; Yousefpour et al., 2011). The combination therapy of this antibody is of prime importance, especially with drugs of taxanes family since both the therapeutic response and survival rate are increased (Sun et al., 2008).

The study is designed for the formulation of paclitaxel-loaded PLGA nanoparticles conjugated with trastuzumab for the effective treatment of breast cancer. Physiochemical characterization, *in vitro* drug release, pharmacokinetic evaluation, and *in vitro* cytotoxicity studies were carried out. The proposed formulations were found safe and effective for the targeting of breast cancer. The developed nanoformulations have the advantage of using polymeric stabilizers which have the potential to improve solubility and enhance stability and bioavailability with no issue of hypersensitivity reactions and are also blocking the pgp efflux transport protein system. The drug delivery in nano-size and surface decoration with the antibody is a unique combination which will not only prevent the particles from being entrapped by the reticuloendothelial systems but also help in accumulation of drug in tumor tissues through EPR (enhanced permeability and retention) effect. So, the therapeutic effectiveness of this drug delivery will be very much improved, and the toxic effects will be minimized. The use of PLGA grade (75:25) results in a more sustained release which has not been used previously with surface conjugation of antibody. Although surface functionalization of paclitaxel nanoparticles has been carried out previously by albumin, polyethylene glycol, and folate, the promising results were obtained in this work in terms of size, stability, drug release profile, *in vitro* cytotoxicity, and pharmacokinetic parameters.

## 2 Materials and Methods

### 2.1 Materials

Paclitaxel (≥99.9% purity) was purchased from Qilu Antibiotic Pharmaceutical Co Ltd China. Poly lactic acid co-glycolic acid (75:25, Resomer^®^ RG 756 H, MW 76000–115000 Da) from Evonik Germany, trastuzumab from Roche Pharmaceuticals United Kingdom, poloxamer 407 and sodium lauryl sulfate (SLS) from Sigma-Aldrich Germany, disodium hydrogen phosphate (Na_2_HPO_4_), dialysis tubing-Dia 27/32”-21.5 mm 30 M MWCO ∼12,000–14,000 Da from Sigma-Aldrich Germany, acetonitrile (purity ≥ 99.9%), and other solvents used were of HPLC grade. The water used for solvent preparation was ultrapure.

### 2.2 Preformulation Studies

#### 2.2.1 Preparation of the Sample

The physical mixtures of drug (paclitaxel) and polymer were prepared (1:1 w/w) with different excipients such as poloxamer (0.5, 1, 1.5, and 2%) and SLS (0.5%). The samples were prepared by simple mixing of drug, polymer, and excipients. The samples were stored for 1 month at 40° ± 2°C and 75 ± 5% RH (Peça et al., 2012). These physical samples were analyzed by FTIR for drug, polymer, and excipients preformulation compatibilities in comparison with nanoformulations.

#### 2.2.2 Compatibility Studies

The interactions between drug (paclitaxel), polymer, and excipients were carried out by preparing binary mixtures. Drug content, physical consistency, and FTIR spectra were examined at each sampling point for any possible drug–excipient incompatibility. The physical interactions among the excipients, drug, and polymer were observed by noting changes in physical consistency.

#### 2.2.3 Determination of the Drug Content Using a UV–Visible Spectrophotometer

The samples containing excipients, excipients and drug, and excipients and polymer were stored under stress conditions and analyzed for determination of the drug content. Samples and standard solutions were dissolved in acetonitrile (ACN) for analysis. The drug content was measured in triplicate.

#### 2.2.4 Fourier Transform Infrared Spectroscopy

An FTIR spectrophotometer was used to analyze the samples for incompatibilities. The samples were prepared by the potassium bromide (KBr) pellet method. Dried potassium bromide was mixed with 1% w/w of the sample and grounded for 3–5 min. The sample was pulverized and converted to a compact mass by compression. The samples were analyzed in the region of 400–4,000 cm^−1^.

### 2.3 Formulation of Plain and Antibody-Conjugated Nanoformulations

Paclitaxel-loaded polymeric nanoparticles were prepared using PLGA as a polymer, poloxamer 407, and sodium lauryl sulfate (SLS) as a stabilizer utilizing the solvent evaporation method (Table 1). PLGA concentration was kept constant (10 mg), while poloxamer 407, SLS, and drug were used in varying concentrations. The developed nanoformulations were characterized for their physicochemical properties [size, polydispersity index (PDI), and zeta potential], drug loading, % entrapment efficiency, and stability. The optimized nanoformulations were then decorated with trastuzumab.

**TABLE 1:** Formulation of paclitaxel with PLGA, 0.05% SLS, and 0.5, 1, 1.5, and 2% poloxamer 407.

S.No	Code	Paclitaxel (mg)	PLGA (mg)	Poloxamer 407	SLS 0.05% (ml)	Time (min)	Temp	Sonication speed (%)
01	PTX 100	1 mg	10 mg	0.5% 5 ml	5 ml	4 min	25°C	99
02	PTX 101	2 mg	10 mg	0.5% 5 ml	5 ml	4 min	25°C	99
03	PTX 102	3 mg	10 mg	0.5% 5 ml	5 ml	4 min	25°C	99
04	PTX 103	4 mg	10 mg	0.5% 5 ml	5 ml	4 min	25°C	99
05	PTX 104	1 mg	10 mg	1% 5 ml	5 ml	4 min	25°C	99
06	PTX 105	2 mg	10 mg	1% 5 ml	5 ml	4 min	25°C	99
07	PTX 106	3 mg	10 mg	1% 5 ml	5 ml	4 min	25°C	99
08	PTX 107	4 mg	10 mg	1% 5 ml	5 ml	4 min	25°C	99
09	PTX 108	1 mg	10 mg	1.5% 5 ml	5 ml	4 min	25°C	99
10	PTX 109	2 mg	10 mg	1.5% 5 ml	5 ml	4 min	25°C	99
11	PTX 110	3 mg	10 mg	1.5% 5 ml	5 ml	4 min	25°C	99
12	PTX 111	4 mg	10 mg	1.5% 5 ml	5 ml	4 min	25°C	99
13	PTX 112	1 mg	10 mg	2% 5 ml	5 ml	4 min	25°C	99
14	PTX 113	2 mg	10 mg	2% 5 ml	5 ml	4 min	25°C	99
15	PTX 114	3 mg	10 mg	2% 5 ml	5 ml	4 min	25°C	99
16	PTX 115	4 mg	10 mg	2% 5 ml	5 ml	4 min	25°C	99

A total of two (2 ml) reconstituted freeze-dried nanoparticles were incubated with trastuzumab at room temperature overnight for surface decoration of antibody on the nanoparticle surface. All the selected paclitaxel nanoformulations were negatively charged, whereas trastuzumab was positively charged (8.457 mV) which resulted in electrostatic attraction between oppositely charged species and trastuzumab. The antibody was easily coated on the surface of paclitaxel-loaded PLGA nanoformulations. The trastuzumab-modified paclitaxel-loaded PLGA nanoparticles were purified by centrifugation at 6,000 rpm at −4°C for 2 min, and 50 µl of trastuzumab (10 mg/ml) was reconstituted with PBS to make up the final volume up to 1 ml (500 μg/ml).

### 2.4 Physicochemical Characterization

#### 2.4.1 Dynamic Light Scattering

The formulations were evaluated for size, polydispersity index **(**PDI), and zeta potential by dynamic light scattering (DLS, at 90° angle and 25°C) using a zetasizer (ZS-90, Malvern Instruments and Malvern, United Kingdom). The surface charge can be determined through zeta potential, i.e., the movements of charged particles in an electric field to predict the stability of colloids. The sample (0.5 ml) of nanoformulation and 1 ml of distilled water were taken, sonicated for 2 min, and placed in cuvettes. An average of three reported values was taken using Malvern software and analyzed statistically (Marsalek, 2014).

#### 2.4.2 Drug Loading and Encapsulation Efficiency

Drug loading efficiency (%, w/w) and drug encapsulation efficiency (%, w/w) of paclitaxel in nanoformulations were determined by centrifugation (15,000 rpm at 25°C for 30 min), followed by UV spectroscopy at 235 nm. The absorbance of the samples was measured, and the % drug loading and % encapsulation were determined by the following formulae (Huang et al., 2007):

$$\text{\% DL}=\frac{\text{Weight}\;\text{of}\;\text{Drug}\;\text{in}\;\text{Nanoparticles}}{\text{Weight}\;\text{of}\;\text{Nanoparticles}}\times 100;$$ (1)

$$\text{\% EE}=\frac{\text{Weight}\;\text{of}\;\text{Drug}\;\text{in}\;\text{Nanoparticles}}{\text{Weight}\;\text{of}\;\text{Drug}\;\text{Feed}}\times 100.$$ (2)

#### 2.4.3 Scanning Electron Microscopy

The morphology of the sample was determined by SEM. The sample was prepared for SEM as per standard protocol in order to make it conducive. The sample was then analyzed for its morphology.

#### 2.4.4 X-Ray Diffraction Study

An X-Ray diffractometer (JDX-3532, Jeol, Japan) was used to carry out XRD patterns of paclitaxel, PLGA, Poloxamer 407, SLS, and paclitaxel nanoformulations. The XRD pattern was determined for its amorphous, semicrystalline, and crystalline nature. The pattern was taken at 3°–40° (2θ).

### 2.5 SDS-PAGE Analysis

After conjugation of antibody on the nanoparticle surface, the structural integrity of trastuzumab on the nanoparticle surface was compared with the native antibody by SDS-PAGE analysis. All the gels were run under reducing conditions using a Mini-PROTEAN^®^ Electrophoresis system (BIO-RAD, United States). It is a technique based on specificity of binding between protein of interest and a probe to allow detection of protein of interest. The protein sample is separated and subjected to a SDS polyacrylamide gel. The sample is transferred electrophoretically from a gel to PVDF membrane. The remaining membrane is blocked by adding a 5% neutral protein (BSA or milk casein) overnight. The membrane is incubated with the primary antibody that is specific to the target protein for 2 h at room temperature. The band containing protein of interest will bind with the antibody. The membrane is then washed to remove the unbound antibody and incubated with the second radioactively labeled antibody for 1 h that binds specifically to the primary antibody–antigen complex which can be visualized on an autoradiograph. The bond will appear dark on the film (Pavlova et al., 2018).

### 2.6 *In Vitro* Evaluation

#### 2.6.1 Drug Release Studies

The dialysis diffusion method was applied for release studies. The membrane having a molecular weight 12,000–14,000 Da was cut in such a way that it can accommodate 2 ml redispersed nanoformulations sealed at both ends. It was then dialyzed against 100 ml of PBS (pH 7.4) in a shaking water bath at 37°C and 60 rpm. At specified time intervals (0.5, 1, 1.5, 2, 3, 4, 5, 6, 7, 8, 10, 12, 24, 36, 48, 72, and 96 h), 2 ml sample was withdrawn and analyzed for drug release. An equal volume of dialyzing media was replaced for each sample. The drug content was determined by using a UV spectrophotometer at 235 nm in each sample. The analysis was conducted in triplicate (Bernkop-Schnürch & Jalil, 2018).

#### 2.6.2 Drug Release Kinetics

The drug release mechanisms were evaluated by applying various release kinetic models (Paarakh et al., 2018).

#### 2.6.3 *In Vitro* Cytotoxicity

*In Vitro* cytotoxicity assay of paclitaxel-loaded polymeric nanoparticles and paclitaxel-loaded polymeric nanoparticles conjugated with trastuzumab antibody and Taxol^®^ was conducted by MTT [yellow tetrazolium salt, 3-(4, 5-dimethylthizol-2-yl)-2, 5, 5-diphenyl tetrazolium bromide] assay using MCF-7 breast cancer cell lines, a widely studied epithelial cancer cell line that has characteristics of differentiated mammary epithelium derived from breast adenocarcinoma (Lee et al., 2015). MCF-7 cell lines of breast adenocarcinoma show moderate overexpression of HER^+2^ and serve as an excellent model for *in vitro* cytotoxic studies (Dhiman et al., 2004). Its hormone sensitivity through expression of estrogen receptor makes it an ideal model for *in vivo* and *in vitro* studies (Holliday & Speirs, 2011). The cells were seeded in a 96-well plate at a density of 1.0 × 10^4^ cells/well and incubated for 24 h at 37°C in 5% CO_2_ at an 85% humidity incubator (Model NU 5700; United States). The medium was replaced after 24 h by paclitaxel-loaded polymeric nanoparticles and paclitaxel-loaded polymeric nanoparticles conjugated with trastuzumab and Taxol^®^ at concentrations ranging from 0.25 μg/ml to 50 μg/ml for 24, 48, and 72 h at 37°C. At specific intervals, the formulations were removed, and 5 mg/ml MTT was added before incubation for 4 h at 37°C. The culture solution was aspirated, and the resulting formazan crystals were dissolved in 100 µl of dimethyl sulfoxide, and the absorbance was measured at 570 nm using a microplate reader (Model FL ×800; Biotek, Winooski, VA, United States). Cytotoxicity was expressed as percentage of cell viability compared to untreated control cells.

$$\text{\% Viability}=\frac{\text{Absorbance of sample}}{\text{Absorbance of control}}\times 100.$$ (3)

### 2.7 *In Vivo* Evaluation

#### 2.7.1 Pharmacokinetic Studies

The New Zealand rabbits weighing 1.5–2.0 kg were purchased from the NIH (National Institute of Health), for *in vivo* pharmacokinetics. The design and study was approved by the Ethical Committee of Pharmacy Department, University of Swabi (Pharm/EC/002). The rabbits were given access to water and food. The animals were excluded by killing/using chloroform anesthesia during the study in case of any distress. The dose at the rate of 2 mg/kg body weight was injected into the marginal ear vein of rabbits, which were divided into two groups for the paclitaxel test and reference formulations. At designated time intervals (10 min, 30 min and 1, 2, 4, 6, 8, 12, 24, 96, and 120 h), blood samples were collected in EDTA tubes and centrifuged at 8,000 rpm for 10 min at 4°C. The Eppendorf tubes were used to collect and store samples at −20°C till analysis. HPLC-UV was used for the analysis of samples.

Various pharmacokinetic parameters such as peak plasma concentration (C_max_), time of peak plasma concentration (T_max_), elimination rate constant (K_el_), elimination half-life (t_1/2_), area under the plasma concentration-versus-time curve (AUC_0-∞_), clearance (Cl), steady state volume (V_ss_), and mean residence time (MRT) were determined using PK-Summit^®^ software.

#### 2.7.2 Statistical Analysis

For the quantification of paclitaxel in samples, mean (X), SD, and %RSD were applied. Comparison between means of treatments was made at *p* ≤ 0.05 using the Student *t* test.

## 3 Results

### 3.1 Preformulation Studies

#### 3.1.1 Drug–Excipients Compatibility Study

**FIGURE 1:**
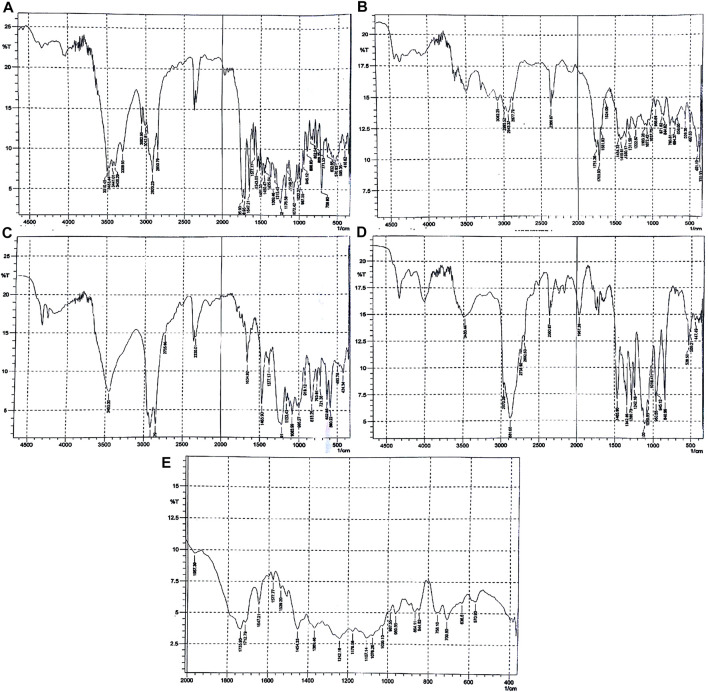
FTIR spectra of **(A)** paclitaxel, **(B)** PLGA, **(C)** SLS, **(D)** poloxamer 407, and **(E)** nanoformulation.

The samples were prepared using binary mixtures of the drug, polymer, and excipients (1:1), stored for 01 months under stress conditions, and inspected visually for any change in color and texture. The drug, polymer, and excipients compatibility study was performed by FTIR at day 1 and 30. The results of FTIR are shown in Table 2 and Figure 1. The concentration of the standard drug and samples at day 1, 15, and 30 were evaluated as given in Table 3.

**TABLE 2:** Result of the drug–excipients compatibility study.

Time	Test	Sample 01	Sample 02	Sample 03	Sample 04	Sample 05	Sample 06	Sample 07	Sample 08	Sample 09
**Day 01**	FTIR spectra	Complies	Complies	Complies	Complies	Complies	Complies	Complies	Complies	Complies
	Physical consistency	ʺ	ʺ	ʺ	ʺ	ʺ	ʺ	ʺ	ʺ	ʺ
**Day 30**	FTIR spectra	ʺ	ʺ	ʺ	ʺ	ʺ	ʺ	ʺ	ʺ	ʺ
	Physical consistency	ʺ	ʺ	ʺ	ʺ	ʺ	ʺ	ʺ	ʺ	ʺ

**TABLE 3:** Result of drug content determination.

Drug content (%)					
Time	Standard drug	Sample 06	Sample 07	Sample 08	Sample 09
Day 01	99.13	99.09	98.99	99.11	99.54
Day 15	99.27	99.03	99.19	99.63	98.17
Day 30	99.63	99.12	97.79	99.07	99.83

**FIGURE 2:**
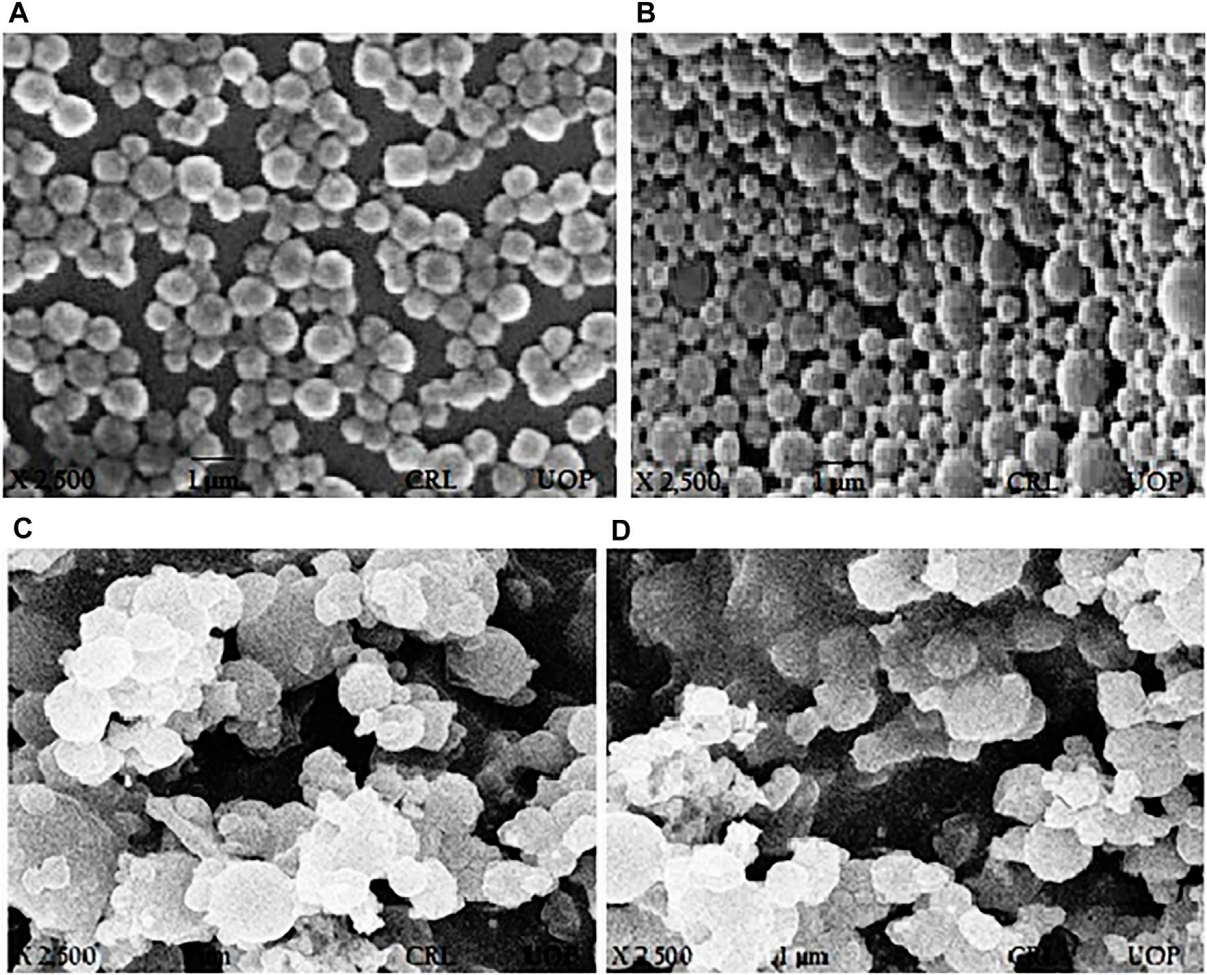
SEM images of paclitaxel-loaded PLGA nanoformulations with poloxamer 407. **(A)** PTX 108, **(B)** PTX 112, **(C)** PTX 108ab, and **(D)** PTX 112ab.

As shown in Figure 1A, the FTIR spectra of paclitaxel show characteristic peaks at 3,441 cm^−1^ (for O-H stretching), 3,309 cm^−1^ (for N-H stretching), aromatic C-H at 2,920–2,850 cm^−1^, peaks at 1708 cm^−1^ for C = O stretching vibration of the ester group, peak at 1,647 cm^−1^ for the amide bond, and peaks at 1,254 cm^−1^ for C-N stretching. The FTIR spectra of PLGA (75:25) showed distinct peaks at 3,200 cm^−1^ for -OH stretching, 2,943 cm^−1^ for -CH stretching, 1751 cm^−1^ for carbonyl –C = O stretching, and at 1,072 cm^−1^ for C-O stretching as given in Figure 2B. The FTIR spectra of poloxamer 407 showed characteristic peaks at 1,111 cm^−1^ and 1,060 cm^−1^ distinguishing of its PEO group and at 2,881 cm^−1^ for CH_2_-CH_2_ stretching as shown in Figure 1C. The FTIR spectra of SLS showed characteristic peaks at 1,219–1,153 cm^−1^ for S-O stretching and at 2,850 cm^−1^ for -CH stretching as shown in Figure 1D.

The characteristic peaks of paclitaxel was not present in the FTIR spectrum which means that the drug is completely encapsulated by the polymer, but the main peaks for PLGA, poloxamer 407, and SLS remain the same indicating the absence of any interaction between the drug, polymer, and stabilizers used as shown in Figure 1E.

### 3.2 Physicochemical Characterization

PTX 108 and PTX 112 were selected for conjugation of the antibody on the basis of particle size, polydispersity index, surface charge, zeta potential, and encapsulation efficiency. The physicochemical properties of the developed nanoformulations before and after antibody conjugation were determined.

#### 3.2.1 Particle Size, PDI, Zeta Potential, and Encapsulation Efficiency

The particle size was within the range of 180 ± 1.22 to 202 ± 36.17 nm for 0.5%, 199 ± 21.80 to 224 ± 26.98 nm for 1%, 202.3 ± 14.5 to 224 ± 26.98 nm for 1.5%, and 229 ± 13.24 to 408 ± 11.27 nm for 2% poloxamer 407. The particle size, PDI, zeta potential, and drug loading and encapsulation efficiency of paclitaxel nanoparticles are given in Table 4. The physicochemical properties and encapsulation efficiency of paclitaxel nanoparticles with or without antibody conjugation are given in Table 5.

**TABLE 4:** Formulation of paclitaxel with PLGA, 0.05% SLS, and poloxamer 407.

No.	Drug: PLGA (mg)	Poloxamer 407 (%)	Size (nm)	PDI	Zeta potential (mv)	(%) Encapsulation efficiency	(%)Drug loading
PTX 100	1:10	0.5	180 ± 1.22	0.11 ± 0.01	−22.1 ± 1.5	64	6.4
PTX 101	2:10	0.5	184.6 ± 1.03	0.13 ± 0.01	−20.1 ± 1.1	45	9.0
PTX 102	3:10	0.5	190 ± 3.48	0.13 ± 0.03	−20.7 ± 1.8	61	18.3
PTX 103	4:10	0.5	202 ± 36.17	0.3 ± 0.01	−19.1 ± 1.5	53	21.2
PTX 104	1:10	1	199 ± 21.80	0.4 ± 0.01	−26.85 ± 0.03	77	7.7
PTX 105	2:10	1	215 ± 18.72	0.6 ± 0.01	−24.1 ± 0.15	65	0.13
PTX 106	3:10	1	304 ± 12.99	0.6 ± 0.04	−26.8 ± 0.23	65	19.5
PTX 107	4:10	1	224 ± 26.98	0.8 ± 0.02	−23.08 ± 0.1	63	25.2
PTX 108	1:10	1.5	202.3 ± 14.5	0.17 ± 0.03	−35.2 ± 0.12	89	8.9
PTX 109	2:10	1.5	215 ± 28.3	0.2 ± 0.03	−34.5 ± 0.03	71	14.2
PTX 110	3:10	1.5	300 ± 17.1	0.19 ± 0.02	−30.25 ± 0.25	65	19.5
PTX 111	4:10	1.5	331 ± 22.5	0.3 ± 0.01	−29.75 ± 0.11	57	22.8
PTX 112	1:10	2	229 ± 13.24	0.2 ± 0.01	−40.4 ± 1.6	84	8.4
PTX 113	2:10	2	312 ± 12.41	0.3 ± 0.02	−39.08 ± 0.6	69	13.8
PTX 114	3:10	2	351 ± 10.49	0.3 ± 0.03	−34.21 ± 1.7	68	20.4
PTX 115	4:10	2	408 ± 11.27	0.7 ± 0.02	−28.11 ± 0.7	47	18.8

**TABLE 5:** Particle size, PDI, and zeta potential of nanoformulations before and after surface modification.

Unconjugated nanoformulations					Conjugated nanoformulations				
**No.**	**Size (nm)**	**PDI**	**ZP (mv)**	**EE (%)**	**No.**	**Size (nm)**	**PDI**	**ZP (mv)**	**EE (%)**
PTX 108	202.3 ± 14.5	0.17 ± 0.03	−35.2 ± 0.12	89%	PTX 108ab	223 ± 11.08	0.42 ± 0.04	−25.7 ± 1.4	88%
PTX 112	229 ± 13.24	0.2 ± 0.01	−40.4 ± 1.6	84%	PTX 112 ab	256 ± 13.52	0.32 ± 0.01	−26.5 ± 0.1	84%

#### 3.2.2 Surface Morphology

SEM was used for determining surface morphology of simple paclitaxel nanoparticles and conjugated paclitaxel nanoparticles as shown in Figures 2A,B and Figures 2C,D, respectively.

#### 3.2.3 XRD Studies

**FIGURE 3:**
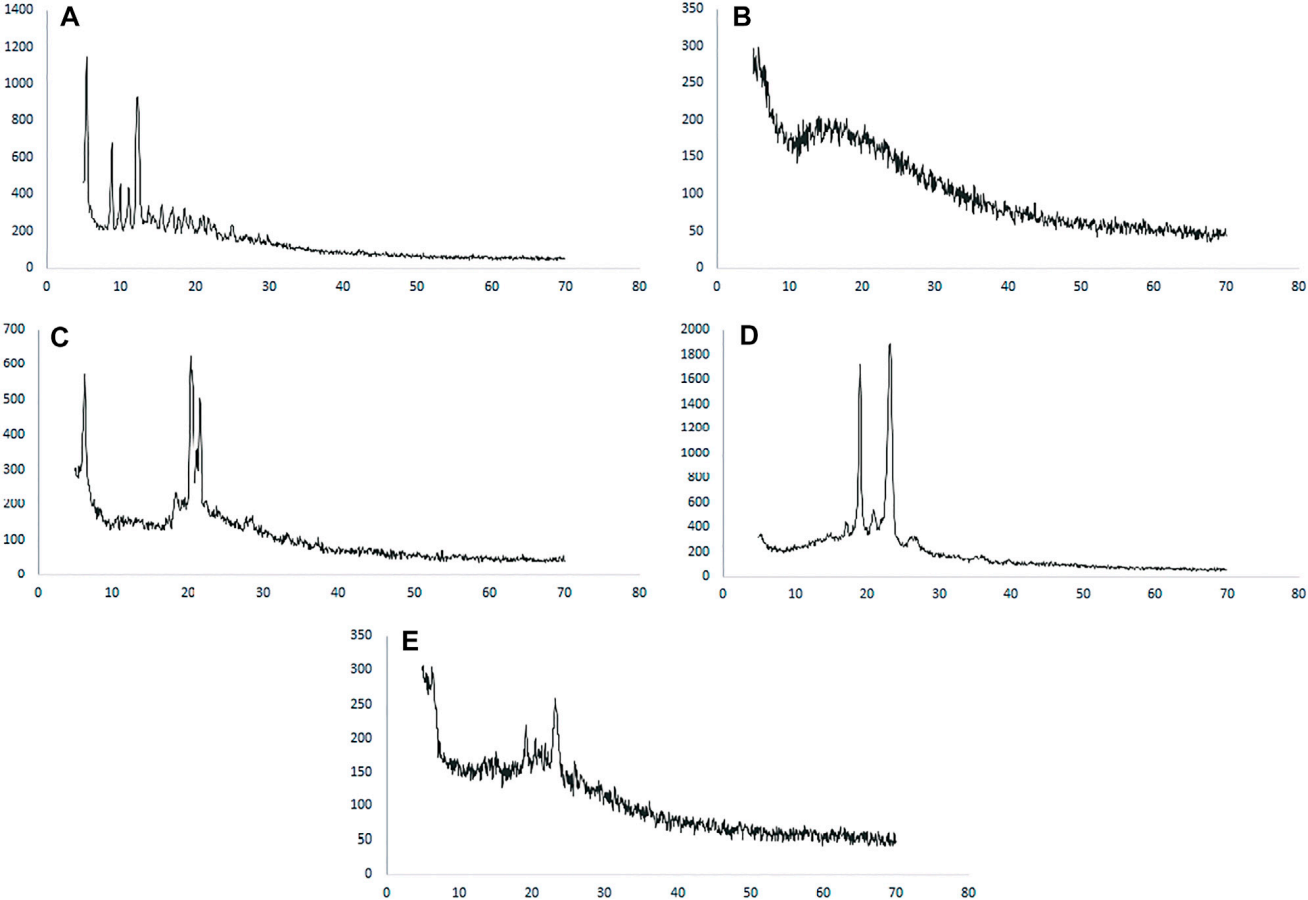
XRD spectra of **(A)** paclitaxel, **(B)** PLGA, **(C)** SLS, **(D)** poloxamer 407, and **(E)** nanoformulation.

The XRD patterns of paclitaxel, PLGA, Poloxamer 407, SLS, and paclitaxel nanoformulations are shown in Figure 3A, in which paclitaxel exhibits several peaks at 2θ value of 5.4°, 8.8°, and 12.25° which shows the crystalline nature of paclitaxel, while no peaks were observed for PLGA which depicts the amorphous nature of the polymer as shown in Figure 3B. The diffractogram of SLS shows two distinct peaks at 2θ value of 20.3° and 21.65° demonstrating the crystalline nature of SLS (Figure 3C), while poloxamer 407 exhibits peaks in the 2θ range at 19.05° and 23.2° as shown in Figure 3D). The XRD pattern of paclitaxel-loaded PLGA nanoformulations exhibits no discrete peaks at any position (Figure 3E).

### 3.3 SDS-PAGE Studies

**FIGURE 4:**
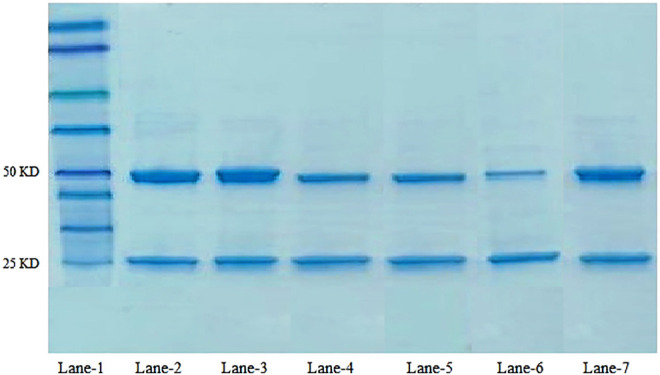
SDS-PAGE of the antibody: lane-1: molecular weight marker; lane 2-3: native antibody trastuzumab; lane-6: PTX 108ab; and lane-7: PTX 112ab.

The structural integrity of trastuzumab after conjugation of the antibody on the nanoparticle surface was confirmed. Under reducing conditions, trastuzumab is detected as two bands of molecular weight 50 KDa and 25 KDa representing heavy and light chains, respectively (Mohamed et al., 2018). An SDS-gel (10%) was ran under reducing conditions as follows: molecular weight marker in lane-1, native antibody in lane-2 and 3, and antibody-conjugated nanoformulations PTX 84ab, PTX 86ab, PTX 108ab, and PTX 112ab in lanes 4,5,6, and 7, respectively, as shown in Figure 4.

### 3.4 *In Vitro* Evaluation

#### 3.4.1 Drug Release Studies

**FIGURE 5:**
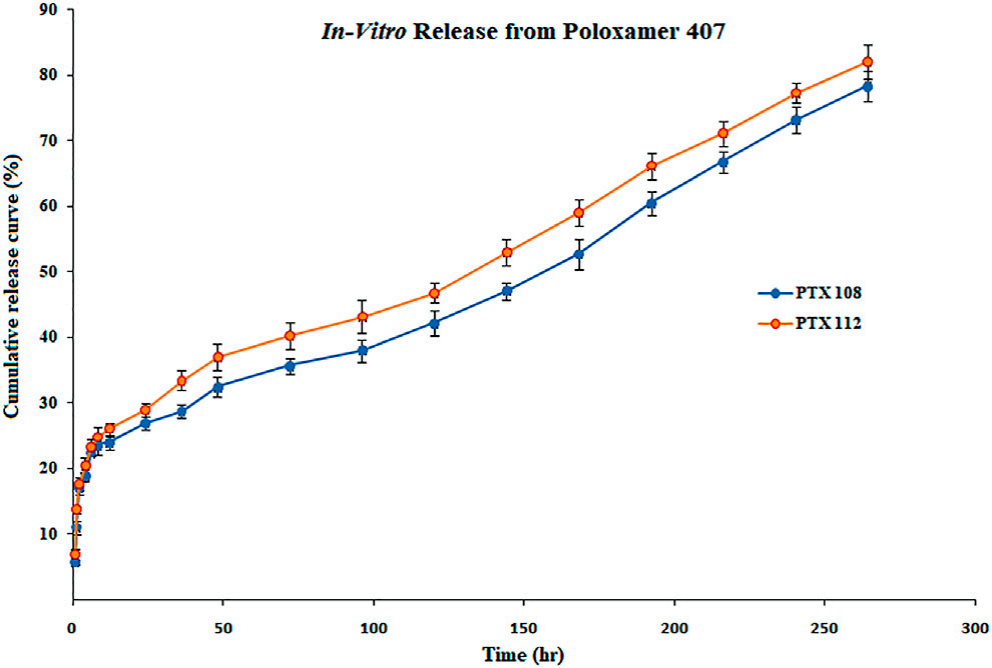
*In vitro* release profile of paclitaxel from nanoformulations of poloxamer 407.

**FIGURE 6:**
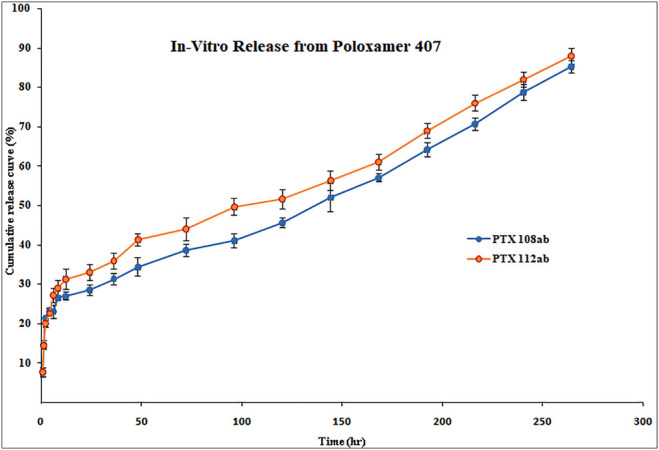
*In vitro* release of Paclitaxel from surface-modified nanoformulations of poloxamer 407.

The *in vitro* release profile of paclitaxel nanoformulations and surface-modified nanoformulations was determined. At specified time intervals (0.5, 1, 2, 4, 6, 8, 10, 12, 24, 36, 48, 72, 96, 120, 144, 168, 192, 216, 240, and 264 h), the samples were withdrawn and analyzed for drug release. All the paclitaxel-loaded PLGA nanoformulations and surface-modified nanoformulations exhibit a bi-phasic release pattern as shown in Figures 5, 6, respectively, which is characterized by an initial burst release in first 24 h followed by a continuous slow release. The initial burst release of paclitaxel from nanoformulations at 24 h was 26 ± 0.23 and 28 ± 0.42% for PTX 108 and PTX 112, while at 264 h drug release was 79 ± 0.09 and 81 ± 0.43% for PTX 108 and PTX 112, respectively. Similarly the initial burst release of paclitaxel from modified nanoformulations at 24 h was 30 ± 0.28 and 33 ± 0.03% for PTX 108ab and PTX 112 ab, while at 264 h, the drug release was 85 ± 0.34 and 88 ± 0.14% for PTX 108 ab and PTX 112 ab, respectively.

#### 3.4.2 *In Vitro* Drug Release Kinetics

Various kinetic models were employed for prediction of drug release mechanisms that include zero-order, first-order, Hixson–Crowell, Korsmeyer–Peppas, and Higuchi. The regression coefficient values (*R* ^2^) obtained and the drug release from PTX 108, PTX 112, PTX 108ab, and PTX 112 ab nanoformulations best fit to the Higuchi model on the basis of higher regression. coefficient (*R* ^2^) values as shown in Table 6.

**TABLE 6:** *In vitro* drug release kinetics of the optimized nanoformulations. Bold values are drug release from nanoformulations best fits to Higuchi model.

Formulation	1st-order	Zero-order	Higuchi	Hixon–Crowell	Korsemeyer–Peppas	n*
	*R* ^2^	*R* ^2^	*R* ^2^	*R* ^2^	*R* ^2^	
PTX 108	0.181	0.8842	**0.9726**	0.8847	0.114	0.5
PTX 112	0.1935	0.8639	**0.9911**	0.8644	0.124	0.5
PTX 108ab	0.3741	0.8588	**0.9919**	0.9222	0.6226	0.5
PTX 112 ab	0.3701	0.8424	**0.9777**	0.9213	0.636	0.5

#### 3.4.3 *In Vitro* Cytotoxicity Studies

**FIGURE 7:**
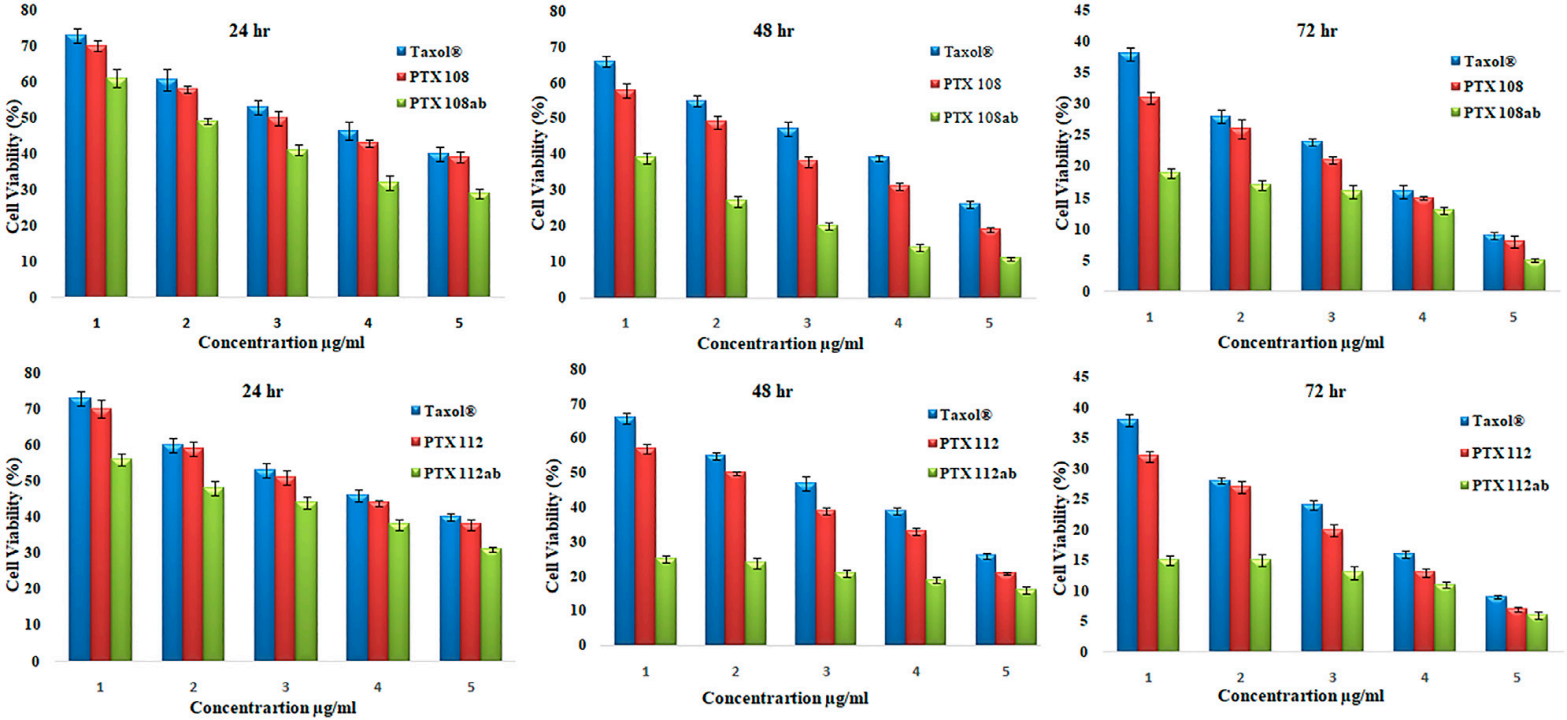
Cell viability (%) of MCF-7 cell lines by Paclixil^®^, paclitaxel-loaded PLGA nanoformulations PTX 108 and PTX 112, and antibody-conjugated paclitaxel-loaded PLGA nanoformulations PTX 108ab and PTX 112ab at 0.25, 2.5, 10, 25, and 50 μg/ml concentration after 24, 42, and 72 h.

The cytotoxicity of the reference drug and paclitaxel-loaded PLGA nanoparticles with and without antibody surface modification was evaluated by performing MTT assay against MCF-7 cells. The MCF-7 cell lines were incubated with Paclixil^®^, paclitaxel-loaded PLGA nanoformulations PTX 112, and antibody-conjugated paclitaxel-loaded PLGA nanoformulations PTX 108ab and PTX 112 ab at 0.25, 2.5, 10, 25, and 50 μg/ml concentration. The cultured cells were analyzed for cell viability at 24, 48, and 72 h. Cytotoxicity as % of cell viability compared to untreated control cells is shown in Figure 7.

### 3.5 *In Vivo* Evaluation

#### 3.5.1 Pharmacokinetic Studies

**FIGURE 8:**
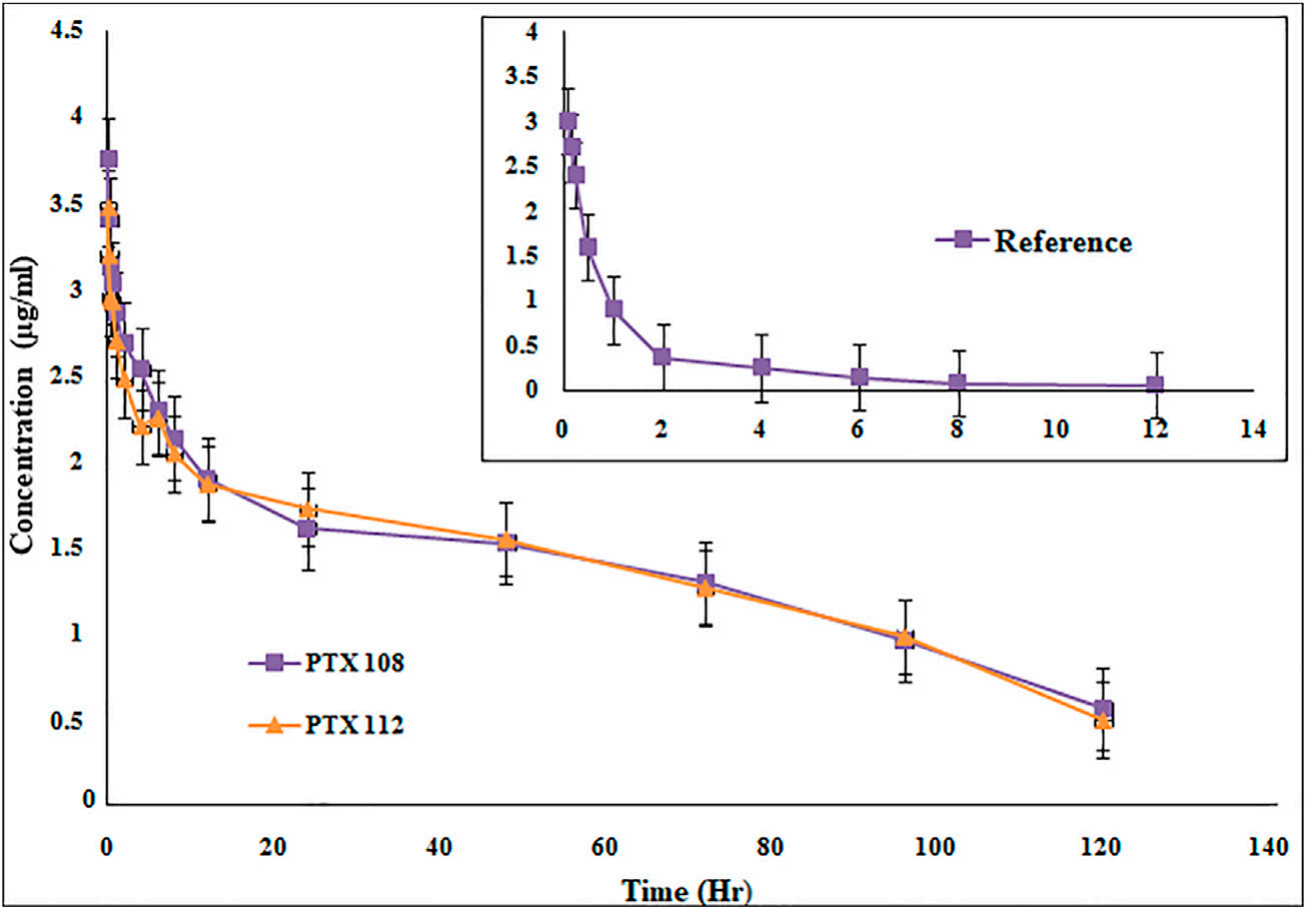
Plasma concentration versus time profile of paclitaxel nanoformulations with poloxamer 407 and SLS.

Rabbits weighing between 1.5 and 2 kg were used as an experimental model for the assessment of various *in vivo* pharmacokinetic parameters of the selected paclitaxel nanoformulations. Selected paclitaxel and its commercially available paclitaxel formulation Paclixil^®^ (reference formulation) were administered (2 mg/kg body weight) *via* the marginal ear vein. For *in vivo* evaluation of selected and commercially available paclitaxel formulation Paclixil^®^, the developed RP-HPLC-UV method was successfully applied (Sakhi et al., 2021). The data were evaluated by non-compartmental analysis using PK-Summit^®^. The results are given in Table 7 and Figure 8.

**TABLE 7:** Pharmacokinetic parameters. The *p*-values are made bold as it shows the significance of results.

Parameter	C_max_	AUC_0-t_	AUMC_**∞**_	MRT	t_½_	V_d_	CL
	μgml^−1^	μghrml^−1^	mghr^2^ml^**−1**^	Hr	Hr	ml	mlh^−1^kg^−1^
Paclixil^®^	3.05 ± 0.78	4.8 ± 0.035	27.2 ± 1.27	5.7 ± 0.14	7.9 ± 0.06	9.4 ± 1.14	7.37 ± 0.5
**PTX 108**	3.75 ± 0.09	194.9 ± 1.04	12433.1 ± 214.1	63.8 ± 1.61	31.2 ± 2.17	65.4 ± 1.98	1.7 ± 1.21
*p-*value	**—**	**—**	**—**	0.001***	0.001***	0.003***	0.001***
**PTX 112**	3.75 ± 0.87	189.4 ± 2.97	10840.2 ± 411.7	57.2 ± 1.16	24.2 ± 3.35	74.5 ± 1.74	1.7 ± 0.69
*p*-value	**—**	**—**	**—**	0.001***	0.002***	0.001***	0.001***

## 4 Discussion

### 4.1 Preformulation Studies

#### 4.1.1 Drug–Excipients Compatibility Study

The stabilizers used for preparing nanoformulations may interact with each other and other active pharmaceutical ingredients which may affect the stability of nanoparticles. Due to change in temperature and humidity, the physical and chemical changes in the dosage form are occurred which can affect stability, biocompatibility, and therapeutic properties of the drug (Chadha & Bhandari, 2014; Patel, et al., 2015). In order to avoid these possible interactions, the drug, polymer, and excipients compatibility study was performed, and the samples were evaluated for drug content, physical consistency, and FTIR spectra. Drug concentration in a dosage form may decrease due to degradation of the drug when stored under stress conditions. Physical and chemical incompatibilities may be triggered by humidity and temperature. The drug contents in the dosage form remained the same throughout the stored period. The IR spectra show no changes in samples at day 1 while showing chemical interaction between drug, polymer, and other drug excipients used in nanoformulations after 30 days (Martins et al., 2014). The FTIR spectrum of the paclitaxel-loaded PLGA nanoparticle showed no characteristic peaks of paclitaxel which means that the drug is completely encapsulated by the polymer, but the main peaks for PLGA, poloxamer 407, and SLS remain same, thus indicating the absence of any interaction between the drug, polymer, and stabilizers used. After visual inspection of samples, no changes in color or physical consistency were noted which indicates the compatibility of drugs and active ingredients with each other.

### 4.2 Physicochemical Characterization

PTX 108 and PTX 112 were selected for having small particle size, high negative zeta potential, and encapsulation efficiency greater than 80% and monodispersed particles. These formulations were further evaluated. The physicochemical properties of nanoformulations before and after antibody conjugation were compared.

#### 4.2.1 Particle Size, PDI, Zeta Potential, and Encapsulation Efficiency

The particle size and PDI change with change in concentration of the stabilizer and amount of the drug in nanoparticle formulations, whereas polymer concentration is kept constant. The size of the nanoparticles increases as the concentration of poloxamer 407 is increased, and there is an increase in PDI with change in stabilizer concentration. Present studies show that the mean particle size increases as the concentration of the stabilizer is increased (Pradhan et al., 2013). This increase in the nanoparticle size is due to excessive adsorption of poloxamer 407 on the nanoparticle surface which results in formation of a thick layer (Redhead et al., 2001). As the stabilizer concentration is increased, viscosity of the aqueous phase increases which results in an increase in particle size by decreasing the net shear stress (Pradhan et al., 2013). As the drug concentration is increased from 1 to 4 mg, there is an increase in the nanoparticle size. This increase in particle size is due to the fact that only a specified amount of the drug can be encapsulated by a constant concentration of the polymer. Any further increase in drug concentration will result in an increase in particle size, thus increasing viscosity of the organic phase (Mu & Feng, 2003; Pradhan et al., 2013). The results show that after the attachment of trastuzumab on the nanoparticle surface there is an increase in size and polydispersity of nanoparticles.

Paclitaxel-loaded polymeric nanoformulations prepared by using poloxamer 407 (0.5, 1, 1.5, and 2%) and SLS (0.05%) show a negative charge, and the zeta potential values decrease as the concentration of the drug is increased from 1 to 4 mg. This decrease in zeta potential is due to an increase in concentration of the drug-to-polymer ratio in the organic phase (Stolnik et al., 2001; Moura et al., 2020). The negative zeta potential was due to the ester and termination group of PLGA chains on the nanoparticle surface (Mu & Feng, 2003) and due to the presence of the anionic surfactant, SLS. As the concentration of poloxamer 407 is increased, there is an increase in zeta potential values. There is an increase in zeta potential values ranging from −19.1 ± 1.5 to −40.4 ± 1.6 mV as the poloxamer 407 concentration is increased from 0.5 to 2% (Redhead et al., 2001; Reddy & Murthy, 2005). High negative potential provides stability as there will be an increase in electrostatic repulsive forces among the nanoparticles which will prevent particle aggregation. The results indicated that the surface charge was shifted to less negative after conjugation of the antibody on the nanoparticle surface due to the positive charge of trastuzumab (Sun et al., 2011a; Sun et al., 2008; P.; Yousefpour et al., 2011).

There is an increase in encapsulation efficiency as the concentration of poloxamer 407 is increased from 0.5 to 2% while keeping PLGA and SLS concentrations constant (Table 4). There is an increase in encapsulation efficiency as the initial concentration of the drug is increased, as more drug molecules are available to interact with the polymer resulting in an increase in encapsulation efficiency. However any further increase in the drug amount will result in saturation of the polymer, leading to a decrease in encapsulation efficiency (Keum et al., 2011; Pradhan et al., 2013). In this work, nanoformulations having encapsulation efficiencies greater than 80% were selected for surface modification which resulted in an increase in size; however, no significant change in encapsulation efficiency was observed with conjugation of the antibody as shown in Table 5.

Surface morphology of nanoparticles determines the circulation time, biodistribution, targeted delivery, and enhanced tumor accumulation as well as cellular uptake of nanoparticles (Truong et al., 2015). The surface of nanoparticles using poloxamer 407 was spherical in shape. After the conjugation of the antibody, the surface of nanoparticles becomes blurry which is due to attachment of the antibody on the surface of nanoparticles and adhesion of nanoparticles (Yousefpour et al., 2011; Mehata et al., 2019).

The XRD pattern of paclitaxel-loaded PLGA nanoformulations exhibits no discrete peaks at any position, so it can be concluded that paclitaxel was completely encapsulated by the polymer and transformed to an amorphous state (Chowdhury et al., 2019; de Oliveira Fortes et al., 2012; Wei et al., 2009).

### 4.3 SDS-PAGE Analysis

The structural integrity of trastuzumab on the nanoparticle surface was compared with the native antibody by SDS-PAGE analysis. As trastuzumab is a protein, when subjected to any type of stress such as preparation process, packaging materials, heating, and agitation, the major response of the monoclonal antibody is aggregation which can result in immunogenic reactions, loss of significant therapeutic activity, denaturation, or inactivation (M Pabari et al., 2013; Mohamed et al., 2018). From the results, it can be observed that trastuzumab shows same behavior after conjugation on the nanoparticle surface as the native antibody which confirms that the integrity of trastuzumab remains the same, and there is no evidence of reduced protein as shown in bands. This validates the feasibility of antibody-decorated paclitaxel nanoparticles for targeting HER^2+^-overexpressed cancer cells.

### 4.4 *In Vitro* Evaluation

All the paclitaxel-loaded PLGA nanoformulations with and without antibody conjugation exhibit a bi-phasic release pattern, which is characterized by an initial burst release in first 24 h followed by a continuous slow release. This slow release is due to the slow degradation of PLGA because the release of paclitaxel from nanoparticles mainly depends on drug diffusion and matrix erosion. The drug that is poorly entrapped/adsorbed on the polymeric matrix results in initial fast release, while the diffusion mechanism is responsible for the slow release of the drug that is localized in the polymeric core of nanoparticles (Fonseca et al., 2002; Pradhan et al., 2013).

It was observed that the drug release from PTX 108, PTX 112, PTX 108ab, and PTX 112 ab nanoformulations best fits to the Higuchi model on the basis of higher regression. coefficient (*R* ^2^) values. The “n” value primarily shows the mechanism of drug release from the polymeric matrix, and it was measured at 60% release concentration. The most common release mechanism followed by these formulations is diffusion followed by erosion. The n value also showed that Fickian diffusion has taken place in the optimized formulations (Costa & Lobo, 2001).

The cytotoxicity studies, as given in Figure 7, shows viability of MCF-7 cells after incubation with Paclixil^®^, paclitaxel-loaded PLGA nanoformulations, and antibody-decorated paclitaxel-loaded PLGA nanoformulations at various concentrations after 24, 48, and 72 h. There is a more effective decrease in cell viability after 72 h than that after 24 and 48 h, which signifies that as the incubation period increases the cellular inhibition increases. The second column in each group in Figure 7 shows viability of MCF-7 cells after treatment with unconjugated paclitaxel nanoformulations, and there is an increase in *in vitro* cytotoxicity as compared to paclitaxel solution. As the concentration of the drug is increased from 0.25 to 50 μg/ml, % viability decreases. The third column in each group shows cellular toxicity of antibody-conjugated paclitaxel-loaded PLGA nanoformulations. There is a significant decrease in % cell viability which indicates that antibody-functionalized nanoformulations are more effective therapeutically than paclitaxel and nanoformulations without antibody conjugation.

It can be depicted from our results that as the concentration of the drug and incubation time increase, cell viability decreases. The surface conjugation of nanoparticles results in an increase in *in vitro* cytotoxicity as compared to nanoformulations without antibody conjugation and Paclixil^®^. Our results are in line with previous data available (Yousefpour et al., 2011; Butt et al., 2012).

### 4.5 *In Vivo* Evaluation

The C_max_, AUC, AUMC, MRT, t_1/2_, and V_d_ have been significantly increased, while Cl has been decreased (Table 7 and Figure 8). The selected formulations were compared statistically with conventional paclitaxel formulation. The plasma concentrations of polymeric nanoformulations were 1.23-fold greater than those commercially available formulation (Guo et al., 2012). The results show 39.38–40.41-fold increase in AUC of polymeric-loaded paclitaxel nanoparticles than that of commercially available paclitaxel. The reported AUC of paclitaxel after administration to rats at a dose of 30 mg/kg were 80.06 ± 5.74 μg-hr/ml for paclitaxel self-microemulsion and 14.61 ± 2.16 μg-hr/ml for paclitaxel solution (Guo et al., 2012). The data suggest that at same concentration, nanoformulations remain in blood for a prolonged period of time and, hence, increase the therapeutic efficacy of the drug. As in nanoformulations, the drug is encapsulated within the hydrophobic polymer which results in sustained release and increase in bioavailability which attributes to an increase in AUC. The other reason of enhanced bioavailability may be due to a decrease in plasma protein binding of polymeric-loaded paclitaxel nanoformulations (Stage et al., 2018). The AUMC_∞_ values of polymeric nanoformulations were significantly greater than those of commercially available formulation.

The MRT of polymeric-loaded paclitaxel nanoparticles is 10.04–11.2-fold than that of the commercially available paclitaxel formulations**.** The reported MRT values of paclitaxel nanoparticles were much higher than that of the pure drug which is in accordance with our results. Polymeric-loaded paclitaxel nanoparticles significantly increase the MRT value by controlling the release of the drug. Drugs formulated in nanoparticles remain in blood circulation for prolonged time due to reduced uptake by the reticuloendothelial system (RES) (Fu et al., 2016). The t_1/2_ of polymeric paclitaxel nanoparticles is 3.06–3.95-fold than that of the commercially available paclitaxel formulations. The V_d_ of polymeric-loaded paclitaxel nanoparticles has increased 6.96–7.93-fold than that of commercially available paclitaxel formulations. The V_d_ of paclitaxel liposome was 0.926 ± 0.057 L and paclitaxel injection was 0.827 ± 0.052 L after IV administration of 3 mg/kg body weight to rabbits (Y. Wei et al., 2014). (Xu et al., 2005). The clearance values of the polymeric nanoformulations decreased than those of the commercially available formulations as reported in the previous literature. The clearance values of paclitaxel liposome was 0.397 ± 0.022 L/h/kg and paclitaxel injection was 0.539 ± 0.038 L/h/kg after IV administration of 3 mg/kg body weight to rabbits (Y. Wei et al., 2014).

The drug eliminates quickly from the systemic circulation after IV administration of paclitaxel injection whereas paclitaxel nanoparticles have shown to improve the pharmacokinetic parameters. The small size of nanoparticles, decreased protein binding, and use of suitable stabilizers result in increased bioavailability of the drug. There is a significant change in pharmacokinetic parameters after encapsulation of paclitaxel in nanoparticles. Paclitaxel-loaded polymeric nanoformulations exhibit an increase in MRT and AUC, while blood clearance is decreased. As the drug remains in blood for a prolonged period of time with nanoparticles, the uptake by the reticuloendothelial system is reduced and uptake of the drug at the target site is enhanced, so improved therapeutic efficacy is achieved with nanoformulations. The use of PLGA grade (75:25) results in a more sustained release which has not been used previously with surface conjugation of the antibody. Although surface functionalization of paclitaxel nanoparticles has been carried out previously by albumin, polyethylene glycol, and folate, however, we got promising results in terms of size, stability, drug release profile, *in vitro* cytotoxicity, and pharmacokinetic parameters in comparison with the reported work (Singla et al., 2002; Nehate et al., 2014).

## 5 Conclusion

Sustained release of paclitaxel-loaded polymeric nanoparticles decorated with trastuzumab was developed using PLGA, SLS, and poloxamer 407 by the solvent evaporation method. The formulations were evaluated for its *in vitro* cellular cytotoxicity against HER^2+^ breast cancer cell lines. The optimized nanoparticles were of particle size less than 300 nm, having a negative charge, and encapsulation efficiency ˃80%. The selected optimized nanoformulations were conjugated with trastuzumab having the desired particle size, PDI, zeta potential, and encapsulation efficiency. SDS-PAGE analyses have shown no evidence of reduced protein, and integrity of trastuzumab remains the same. Scanning electron microscopy (SEM) results have shown that the surface of nanoparticles before antibody conjugation were smooth and spherical, while after the conjugation of the antibody, the surface became blurred which is due to attachment of the antibody on the surface of nanoparticles. The drug release from antibody-conjugated nanoparticles was rapid as compared to unconjugated nanoparticles due to rough surfaces of nanoparticles.

The pharmacokinetic parameters of paclitaxel-loaded polymeric nanoformulations exhibit an increase in MRT, AUC, t_1/2_, and V_d_, while Cl was decreased as compared to those of commercially available paclitaxel nanoformulation. The results of cytotoxicity studies have shown a significant decrease in cell viability as the drug concentration and incubation time increase. The surface conjugation of nanoparticles resulted in greater *in vitro* cytotoxicity than nanoformulations without antibody conjugation and conventional paclitaxel formulations.

## Data Availability

The original contributions presented in the study are included in the article/Supplementary Materials, further inquiries can be directed to the corresponding authors.
